# Ecotoxicity Evaluation of Pure Peracetic Acid (PAA) after Eliminating Hydrogen Peroxide from Commercial PAA

**DOI:** 10.3390/ijerph17145031

**Published:** 2020-07-13

**Authors:** Ravi Kumar Chhetri, Silvia Di Gaetano, Andrea Turolla, Manuela Antonelli, Henrik Rasmus Andersen

**Affiliations:** 1 Department of Environmental Engineering, Technical University of Denmark, 2800 Kongens Lyngby, Denmark; rakc@env.dtu.dk; 2 EIM—Ecological Integrated Management S.R.L., 24124 Bergamo, Italy; silvia.digaetano@mail.polimi.it; 3 Environmental Division, Department of Civil and Environmental Engineering (DICA), Polytechnic University of Milano, 20133 Milano, Italy; andrea.turolla@polimi.it (A.T.); manuela.antonelli@polimi.it (M.A.)

**Keywords:** peracetic acid, hydrogen peroxide, disinfection, ecotoxicity

## Abstract

In recent years, peracetic acid (PAA) has gained a lot of attention as an alternative disinfectant to chlorine-based disinfectants in the water industry. Commercial PAA solutions contain both PAA and hydrogen peroxide (HP), and the degradation of HP is slower than PAA when it is used for disinfection. All previous toxicity studies have been based on commercial PAA, and variance in toxicity values have been observed due to different PAA:HP ratios. In this study, the ecotoxicity of pure PAA was studied, eliminating HP from the commercial PAA mixture using potassium permanganate. Ecotoxicity data were obtained by conducting a battery of ecotoxicity tests: bioassays using *Vibrio fischeri (V. fischeri), Daphnia magna (D. magna)*, and *Pseudokirchneriella subcapitata (P. subcapitata)*. The effect concentration (EC_50_) of pure PAA was 0.84 (a 95% confidence interval of 0.78–0.91) mg/L for *V. fischeri* and 2.46 (2.35–2.58) mg/L for *P. subcapitata*, whereas the lethal concentration (LC_50_) was 0.74 (0.55–0.91) mg/L for *D. magna*. Compared to this, our previous study found that the EC_50_ values of commercial PAA towards *V. fischeri* and *P. subcapitata* were 0.42 (0.41–0.44) and 1.38 (0.96–1.99) mg/L, respectively, which were lower than pure PAA, whilst the LC_50_ for *D. magna* was 0.78 (0.58–0.95) mg/L. These results showed that pure PAA was less toxic to the most commonly used aquatic species for toxicity tests compared to commercial PAA, except for *D. magna*.

## 1. Introduction

Various disinfectants are used in the water industry to reduce the number of pathogenic organisms and eventually inhibit the spread of diseases. Chlorine-based disinfectants, such as hypochlorite and chlorine dioxide, could be used to reduce contamination by microorganisms [1]. However, due to the fact that both the ecotoxicity and disinfection by-products (DBPs) of these compounds are of environmental concern, the use of these compounds is strongly discouraged [2,3,4,5]. Organic peroxides, such as peracetic acid (PAA), have already been used as alternatives to chlorine-based disinfectants for wastewater over the last few decades [6,7,8,9], showing both a lower ecotoxicity and potential of DBP formation [10]. Recently, PAA was also used for the disinfection of combined sewer overflows [11] and in aquaculture [12,13]. 

PAA is a powerful disinfectant with a wide spectrum of antimicrobial activity. Commercial PAA (cPAA) is a quaternary equilibrium mixture of PAA, acetic acid, and hydrogen peroxide (HP) (Equations (1) and (2)):

(1) $$CH_{3}COOH+H_{2}O_{2}⇌CH_{3}COOOH+H_{2}O$$

(2) $$CH_{3}CO-OOH+2e^{-}\to CH_{3}CO-O^{-}+HO^{-}$$

The residues formed after cPAA use are acetic acid, hydrogen peroxide, oxygen, and water. The degradation of hydrogen peroxide is slower than PAA [14,15], and it has a stringent discharge limit to surface water in Denmark [16]. Therefore, it is important to evaluate the ecotoxic effect of residual disinfectant, meaning PAA and HP, to assess the potential impact of discharged disinfected effluents in receiving waters and related aquatic ecosystems. There are various commercial PAA formulations available in the market consisting of diverse PAA:HP ratios. As an example, commercial PAA MinnFinn (Minntech Corporation, Minneapolis, MN, USA) consists of 4.5% *w/w* PAA and 22% *w/w* HP, Wofasteril E400 (Kesla Pharma Wolfen GmbH , Greppin, Germany) consists of 40% *w/w* PAA and 12% *w/w* HP, and PAA from Solvay Chemie, Milano, Italy consists of 15% *w/w* PAA and 23%w/w HP [17,18]. The diverse PAA:HP ratios in cPAA potentially leads to different disinfection efficiencies and toxicities at the same cPAA concentration due to the different concentrations of HP present in the mixture. Due to this, the ecotoxicity data of cPAA reported in the literature are not similar and difficult to compare [17,18]. Usually, the ecotoxicity of cPAA and hydrogen peroxide is studied by adopting *Vibrio fischeri* (*V. fischeri*), recently classified as *Aliivibrio fischeri*, *Daphnia magna* (*D. magna*), and *Pseudokirchneriella subcapitata* (*P. subcapitata*) as indicators. To our knowledge, the ecotoxicity data and degradation kinetics of pure PAA (pPAA), meaning a PAA solution without hydrogen peroxide, are not available to interpret the role of these two chemicals in affecting aquatic ecosystems.

The aim of this study was to compare the ecotoxic effects of pPAA and cPAA after eliminating hydrogen peroxide from cPAA. The ecotoxic effect was studied with bioassays using *V. fischeri*, *D. magna*, and *P. subcapitata*. Moreover, the decay kinetics of pPAA on the test medium was evaluated to account for the fact that PAA’s concentration decreases over time, depending on water constituents.

## 2. Materials and Methods

### 2.1. Chemicals and Chemical Analyses

ABTS (2,2″-azino-bis (3-ethylbenzothiazoline-6-sulfonic acid) diammonium salt), potassium permanganate, and a commercial PAA solution containing 30–40% *w/w* PAA, 40–45% *w/w* acetic acid, and 5% *w/w* HP of technical grade was purchased from Sigma-Aldrich (Brøndby, Denmark). 

Hydrogen peroxide was removed from the cPAA solution by titration with potassium permanganate (KMnO_4_). The endpoint of titration was determined by the slight appearance of the pink color of permanganate when hydrogen peroxide was removed from the cPAA. 

### 2.2. Peracetic Acid Concentration Profiles

Concentration profiles of pPAA in the *Daphnia magna* and *P. subcapitata* test mediums were obtained by measuring PAA concentration over time. The decay of pPAA was measured at 0, 18, 24, 48, and 72 h in the media, i.e., the same time points for which the mortality of *D. magna* was recorded, and algal biomass was extracted to calculate the growth inhibition of *P. subcapitata*. PAA concentration was analyzed using the colorimetric method described by Chhetri et al. [14,19] based on the selective oxidation of ABTS by PAA without interference from hydrogen peroxide. The limit of detection (LOD) of PAA was 0.04 mg/L, and the limit of quantification (LOQ) of PAA was 0.05 mg/L.

Different concentrations of pPAA—0.16, 0.3, 0.63, 1.25, 2.5, 3, and 6 mg/L—were used to obtain concentration profiles. However, data from the 0.16–0.3 mg/L pPAA profiles are not presented because concentration measurement at 18 h was lower than the LOQ. 

### 2.3. Bioassays

For each inhibition test, five pPAA concentrations were tested in two types of experiments: a range finding test and a final test. For the range finding experiments, 0.03–10 mg/L of pPAA were used, and for the final experiment, 0.03–2.5 mg/L of pPAA were used.

#### 2.3.1. Microbial Toxicity

The commercial BioTox^™^ (AboatoxOy, Masku, Finland) assay kit was used to measure the microbial toxicity towards the photobacterium *V. fischeri*. The tests were carried out in accordance with the ISO 11348-3 [20] test method. The pH of all samples was adjusted to 7.0 ± 0.2 by using 1 M NaOH or 1 M H_2_SO_4_ solutions prior to the assay. The final chloride concentration of 20 g/L (2% *w/v*) in the samples was obtained by adding NaCl. After mixing 100 µL of the test solution with 100 µL of the luminescent bacterial suspension, light emission was measured after 5, 15, and 30 min of contact time in duplicate (in two separate vials) at a temperature of 15 °C. Relative inhibition at 5, 15, and 30 min was calculated on the basis of controls to which no test compound was added.

#### 2.3.2. Crustaceans Immobilization Test

To test the immobilization tests with the crustacean *D. magna,* the method and testing conditions prescribed by ISO 6341 [21] were used. Tests with *D. magna* neonates (less than 24 h old) were carried out at 20 ± 2 °C in the dark. For each tested pPAA concentration, immobilization tests were carried out in four replicates. For the control, four beakers without the addition of any testing chemical were tested. In each replicate, 25 mL of the testing solution was placed in 100 mL glass beakers, and five neonates were added. The number of immobile *D. magna* neonates was counted after 24 and 48 h of incubation with the test solutions. Animals were counted as dead if they remained settled at the bottom of the test container and did not swim within 15 s of observation. The mortality values were calculated as the percentage of dead *D. magna* neonates compared to the initial number of animals added in each replicate. The control group was used to ensure that no mortality occurred in beakers without the addition of the test compound.

#### 2.3.3. Algal Growth Inhibition Test

The toxicity towards the freshwater microalgae *P. subcapitata* was tested using the modified ISO 8692 [22] test method. A laboratory culture of *P. subcapitata* was obtained from the Norwegian Institute for Water Research, Oslo, Norway (NIVA). pPAA concentrations were inoculated with exponentially growing algae to a density of 10^4^ cells/mL. For each tested pPAA concentration, algal tests were performed in triplicate. For the control, four beakers without the addition of any testing chemical were tested. A mini-scale test with 4 mL of test solution in 30 mL polystyrene containers (NUNC, Roskilde, Denmark) was applied in this study [23]. The containers were placed on a shaker (200 rpm) at 20 ± 1 °C to allow for mixing and CO_2_ diffusion. The containers were continuously illuminated at 80–105 µE/m^2^/s (measured under the test vessel) using a cold light fluorescent tube emitting light in the visible spectrum. Light intensity in the test setup was measured using a LI-COR light meter (model LI-189, LI-COR Biosciences, Lincoln, NE, USA) with an attached quantum sensor, measuring light within the wavelength range of 400–700 nm. The tests were performed at a pH of 7.8–8.0 with typical control growth rates of 1.4–1.6 d^−1^ during the 72-h incubation. Samples of 0.4 mL were taken at times of 0, 24, 48, and 72 h. The algal growth rates were calculated based on the total algal biomass in each sample quantified by acetone extractions of chlorophyll, as described by Mayer et al. [24]. The fluorescence of the samples was subsequently measured at an excitation wavelength of 430 nm and an emission wavelength of 671 ± 20 nm using a fluorescence spectrophotometer (Hitachi F-2000, Hitachi High-Technologies Corporation, Tokyo, Japan).

#### 2.3.4. Statistical Analyses of Bioassays

A logistic regression (Logit) model was used for estimation of effect concentrations (EC) with 95% confidence intervals for the inhibition of *V. fischeri*. The calculation of acute toxicity of *D. magna* was done using ToxCalc™ v5.0 program (Tidepool Scientific Software, McKinleyville, CA, USA). Lethal concentrations (LC) with 95% confidence intervals were calculated using the Probit model along with linear regression by maximum-likelihood estimation (Tidepool Scientific, Tidepool Scientific Software, McKinleyville, CA, USA). To estimate the growth rates of *P. subcapitata* and concentration–response curves, a nonlinear-regression program with control variance weighting [25] assuming a lognormal distribution was used. By the use of logistic curve fitting and inverse estimation, EC-values were determined with corresponding 95% confidence limits.

## 3. Results and Discussion

### 3.1. Preparation of Pure PAA

Hydrogen peroxide from cPAA was removed by titration with potassium permanganate to obtain pure PAA. To find the optimal permanganate dose to remove the hydrogen peroxide from cPAA, different concentrations of permanganate were used for titration (Table 1). After titration, the removal of hydrogen peroxide from commercial PAA was studied. In addition, the loss of PAA concentration from commercial PAA was also studied. Moreover, the reappearance of hydrogen peroxide in the pPAA stock solution was studied for 48 h. The optimal dose of 31 mg/L KMnO_4_ removed 99% of hydrogen peroxide from cPAA; however, 7% of PAA was also removed during titration. The reappearance of hydrogen peroxide was not observed in the pure PAA solution until 48 h.

### 3.2. Toxicity Values

**Figure 1:**
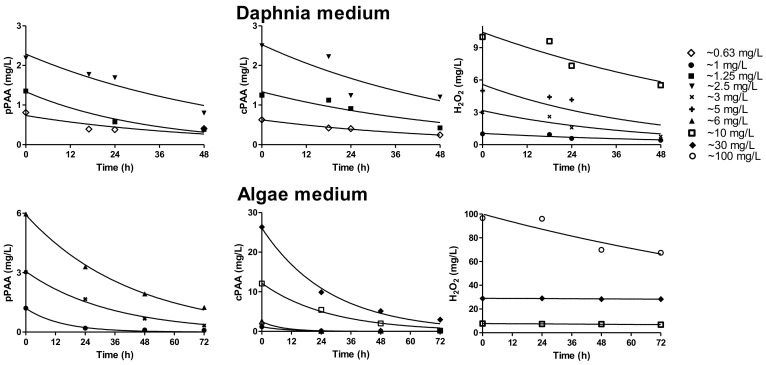
Concentration profiles of pPAA in the *Daphnia* test medium and the algal test medium. Concentration profiles of commercial PA (cPAA) and hydrogen peroxide in a *Daphnia* test medium and an algal test medium were derived from Chhetri et al. [26,27]. The fitted curve was based on the first-order degradation kinetics model presented in Equation (3).

The ecotoxicity data of pPAA on *V. fischeri, D. magna*, and *P. subcapitata* are reported in Table 2. Concentration–response curves corresponding to the effect concentration and lethal concentration values are presented in the Supplementary Materials (see Supplementary Materials Figures S1–S3). Effect concentration and lethal concentration values were calculated using three different approaches. The first approach was based on initial pPAA concentration, not taking into account the pAA decay during contact time. The second approach was done by calculating the median concentration of pPAA over the test duration from the concentration profiles of pPAA in the algal and *Daphnia* test mediums presented in Figure 1. In the third approach, EC_50_ and LC_50_ values were referred to the area under the curve (AUC) describing the concentration profile of pPAA. The effect concentration and lethal concentration were lower when they were calculated using median concentration compared to nominal concentration and AUC. Among the three aquatic species, pPAA was most toxic to *D. magna* and *V. fischeri*. The toxicity (EC_50_) of pPAA towards *V. fischeri* was 0.84 mg/L, whilst the toxicity from cPAA towards *V. fischeri* was 0.42 mg/L [26]. Moreover, the toxicity (EC_50_) of pPAA towards *P. subcapitata* was 2.46 mg/L, which was twice that of cPAA (1.38 mg/L) [27]. The toxicity data of pPAA obtained in this study were different than the toxicity data of cPAA reported in the literature. In the ECETOC (The Centre for Chemical Safety Assessment) report, the EC_50_ values of PAA on *P. subcapitata* were reported as <1 mg/L [28], whilst Antonelli et al. [17] found an EC_50_ value of 8.89 mg/L. The LC_50_ values of cPAA on *D. magna* reported by ECETOC [28] ranged from 0.35 to 1.1 mg/L. Liu et al. [18] reported a *D. magna* LC_50_ range from 0.18 to 0.77 mg/L, and Antonelli et al. [17] found an LC_50_ value of 0.15 mg/L. The *V. fischeri* EC_50_ of cPAA reported by Antonelli et al. [17] was 0.16 mg/L, which was six times lower than pPAA observed in this study. Hence, literature data on the aquatic toxicity of PAA are sparse and somewhat dissimilar [10]. The difference in the toxicity values might have been due to the diverse PAA:HP ratios that were present in the cPAA used for this toxicity study.

Both *V. fischeri* and *P. subcapitata* were less sensitive to pPAA when hydrogen peroxide was removed from the cPAA. Nevertheless, the toxicity of pPAA towards *D. magna* was almost identical to the one observed using cPAA [26]. *D. magna* has a different cell structure and morphology than *V. fischeri* and *P. subcapitata*, and this alters the toxic mechanism of PAA towards these aquatic species. The PAA mixture with a lesser hydrogen peroxide concentration displayed a low toxicity towards *D. magna* when cPAA solutions with diverse PAA:HP ratios were used [18]. Hydrogen peroxide exhibits toxicity towards the aquatic species, but the inhibition occurs at higher values: EC_50_ values of 5.67 mg/L for *V. fischeri* and 2.90 mg/L for *P. subcapitata*, as well as an LC_50_ value of 3.46 mg/L for *D. magna*, have been found [26], which are higher than the values found for pPAA and cPAA here. The results showed that when hydrogen peroxide was eliminated from cPAA, there was a reduction of the toxicity towards the aquatic species, though to different extents. The EC_50_ values of cPAA on *V. fischeri* and *P. subcapitata* were nearly half the values for pPAA, thus suggesting a higher toxicity. A possible reason for this might have been the synergic effect of hydrogen peroxide present in the cPAA. The two stages of an attacking scheme of PAA to bacteria was proposed by Flores et al. [29] when PAA is used for disinfection. PAA eliminates some specific components (e.g., catalase enzyme) from the cells of microorganisms in the first step, which would otherwise inhibit the parallel action of hydrogen peroxide. However, when hydrogen peroxide was eliminated from cPAA, the synergic effect of hydrogen peroxide was also eliminated, as was observed experimentally by comparing the toxicity values of PAA and cPAA. Toxicity values (EC_50_/LC_50_) less than 1 mg/L provide a classification “acute toxic,” i.e., very toxic to the aquatic organisms according to CLP (Classification, Labeling and Packaging) regulation [30]. PAA without hydrogen peroxide is considered very toxic towards *V. fischeri* and *D. magna* according to CLP regulation; however, this is not the case for *P. subcapitata*. 

### 3.3. Concentration Profiles in Test Media

Generally, static ecotoxicity tests must be carried out under stable exposure conditions, i.e., concentrations are maintained within 80–120% of the nominal concentration throughout the test period [31]. In the literature, measured concentrations do not seem to have been reported, and compound degradation (prior to or during testing) was not considered. Therefore, the concentration profiles of pPAA in the *Daphnia* and algal test mediums were obtained by measuring concentration over time in this study (Figure 1). One of the purposes of measuring the concentration profiles was to evaluate the decay kinetics of pPAA during the period when toxicity was tested. Moreover, the concentration profiles of pPAA and cPAA in the *Daphnia* and algal test mediums were studied to compare the degradation kinetics. Finally, a comparison of the degradation of pPAA was done with the degradation of cPAA on wastewater effluent and combined sewer overflow (CSO). A first order degradation kinetics model described in Equation (3) was used for the curve fitting in Figure 1, with derived parameters presented in Table 3.

(3) $$C_{t}=C_{0}\cdot e^{-kt}$$

In Equation (3), $C_{t}$ (mg/L) is the residual disinfectant concentration at time t, $C_{0}$ is the applied disinfectant concentration (mg/L), k is the rate constant (h^−1^), and t is contact time (h).

The degradation of pPAA was fast in the algal medium compared to the *Daphnia* test medium. The half-life of 3 mg/L of pPAA in the algal test medium was 22.9 h, which was similar to 1.3 mg/L of pPAA in the *Daphnia* test medium (22.9 h) (Table 3).

The degradation of pPAA in the test medium was similar to our previous study, where the degradation of cPAA was studied in wastewater effluent and combined sewer overflows [14,32]. The residual concentration of the disinfectants derived from the concentration profiles can be used to assess the potential impact of disinfected effluents in receiving waters and related aquatic ecosystems. The data from ecotoxicity test and residual pPAA concentration obtained from the concentration profiles are important for the indicative environmental risk assessment to estimate the predicted no effect concentration (PNEC) [33]. The PNEC_freshwater_ values of pPAA were calculated by dividing the lowest EC_50_ or LC_50_ value by assessment factors selected by referring to the Technical Guidance Document [33]. An assessment factor of 1000 was selected, as only data from short-term toxicity tests at three different trophic levels were available. For setting environmental quality standards, PNEC values of pPAA (0.74 µg/L) could be used with additional considerations of the potential for bioaccumulation and the persistency of the compounds. The log Kow value of PAA at pH 7 was −1.57, which is lower than 3. Therefore, PAA does not have the potential for bioaccumulation, and so the risk of the secondary poisoning of predators in an aquatic ecosystem is very low. PAA is not expected to be persistent in an aquatic environment due to the fast degradation and short half-lives shown in Table 3.

## 4. Conclusions

This study presented ecotoxicity data on pure PAA on three trophic levels in aquatic ecosystems. A disinfectant solution of pure PAA without hydrogen peroxide was obtained via the selective destruction of hydrogen peroxide by potassium permanganate. The results of toxicity tests revealed that pPAA was less toxic to *V. fischeri* and *P. subcapitata* compared to commercial PAA. Pure PAA and commercial PAA showed similar toxicity to *D. magna*. The slow degradation of pure PAA on *Daphnia* and algal test mediums was observed, and it was similar to commercial PAA. This highlighted that pure PAA is less toxic to aquatic ecosystems when hydrogen peroxide is removed and confirmed its potency as an alternative to commercial PAA for disinfection.

## Figures and Tables

**Table 1:** Removal of peracetic acid (PAA) and H_2_O_2_ after titration with different concentrations of KMnO_4_ (mean ± standard deviation, *n* = 3).

KMnO_4_ (mg/L)	PAA (mg/L)	HP (mg/L)	Removal-PAA	Removal-HP
0	1160	150	0%	0%
16	1148	6	1 ± 2%	96 ± 0.6%
31	1079	1.5	7 ± 1%	99 ± 0.2%
62	974	1.5	16 ± 1%	99 ± 0.1%
92	835	1.5	28 ± 3%	99 ± 0.1%

HP: hydrogen peroxide.

**Table 2:** Effect concentration (EC_10_ and EC_50_) on *V. fischeri* at 30 min contact time of pure PAA (pPAA) and algae at 72 h of contact time of pPAA and lethal concentration (LC_10_ and LC_50_) on *Daphnia magna* at 48 h of contact time of pPAA based on PAA’s nominal concentration (95% confidential interval in parenthesis). Number of data available for calculations: 5 for each indicator.

Test Organism		pPAA Nominal Concentration (mg/L)	pPAA Median Concentration (mg/L)	Area under the Curve (mg × min/L)
*Vibrio fischeri*	EC_10_	0.47 (0.38–0.58)	N/A	N/A
	EC_50_	0.84 (0.78–0.91)	N/A	N/A
*Pseudokirchneriella subcapitata*	EC_10_	1.43 (1.25–1.63)	0.30 (0.22–0.41)	36.8 (29.5–45.9)
	EC_50_	2.46 (2.35–2.58)	0.88 (0.83–0.94)	77.2 (70.6–84.3)
*Daphnia magna*	LC_10_	0.45 (0.20–0.59)	0.32 (0.18–0.38)	17.4 (8.7–21.1)
	LC_50_	0.74 (0.55–0.91)	0.43 (0.36–0.49)	24.7 (19.6–28.3)

N/A = Not analyzed.

**Table 3:** Fitted parameters of concentration profiles of pPAA, cPAA, and HP in the *D. magna* (*n* = 4) and algal test media (*n* = 5) shown in Figure 1. The latter two are from Chhetri et al. [26,27].

Assay Medium	Disinfectants	Nominal Concentration (mg/L)	C_initial_ (mg/L)	*k* × (h^−1^)	*R* ^2^	*t*_½_ (h)
*Daphnia*	pPAA	0.6	0.7	2.1 × 10^−2^	0.67	33
		1.3	1.3	3.0 × 10^−2^	0.98	23
		2.5	2.3	1.8 × 10^−2^	0.91	40
	cPAA	0.6	0.6	2.0 × 10^−2^	0.99	35
		1.3	1.3	1.8 × 10^−2^	0.87	38
		2.5	2.5	1.7 × 10^−2^	0.75	40
	HP	1	1.0	1.8 × 10^−2^	0.81	39
		3	3.2	2.4 × 10^−2^	0.86	29
		5	5.6	2.3 × 10^−2^	0.71	30
		10	10.4	1.2 × 10^−2^	0.85	57
Algae	pPAA	1	1.2	6.9 × 10^−2^	0.99	10
		3	3.0	2.8 × 10^−2^	0.98	24
		6	5.9	2.3 × 10^−2^	0.99	30
	cPAA	1	1.2	9.9 × 10^−2^	0.98	7
		2	2.5	1.2 × 10^−1^	0.99	6
		10	12.2	3.7 × 10^−2^	0.99	19
		30	26	3.6 × 10^−2^	0.99	19
	HP	10	7.8	1.8 × 10^−3^	0.94	394
		30	29	3.4 × 10^−4^	0.71	2000
		100	100	5.6 × 10^−3^	0.83	122

## Supplementary Materials

The following are available online at https://www.mdpi.com/1660-4601/17/14/5031/s1, Figure S1: Dose response curve of *V. fischeri* from pure PAA at 5, 15, and 30 min exposure time. Dashed lines are 95% confidence interval, Figure S2: Dose response curve of *D. magna* from pure PAA at different exposure time. Blue lines are 95% confidence interval. Figure S3: Dose response curve of *P. subcapitata* from pure PAA at 72 h exposure time. Dashed lines are 95% confidence interval.
