# Supplementary material for: Ecotoxicity Evaluation of Pure Peracetic Acid (PAA) after Eliminating Hydrogen Peroxide from Commercial PAA
Source: Int J Environ Res Public Health. 2020 Jul 13;17(14):5031. doi: 10.3390/ijerph17145031 (PMC7400010; doi:10.3390/ijerph17145031)
Supplement: Supplementary file 1 [file ijerph-17-05031-s001.pdf]

Supporting information

Ecotoxicity evaluation of pure peracetic acid (PAA) after eliminating hydrogen peroxide from commercial PAA

Ravi Kumar Chhetri[1], Silvia Di Gaetano[2], Andrea Turolla[3], Manuela Antonelli[3], Henrik Rasmus Andersen[1]

[1] Department of Environmental Engineering, Technical University of Denmark, Bygningstorvet, Building 115, 2800 Kgs. Lyngby, Denmark; rakc@env.dtu.dk

[2] EIM—Ecological Integrated Management S.R.L., 24124 Bergamo, Italy; silvia.digaetano@mail.polimi.it

[3] Environmental Division, Department of Civil and Environmental Engineering (DICA), Polytechnic University of Milano, 20133 Milano, Italy; andrea.turolla@polimi.it (A.T.); manuela.antonelli@polimi.it (M.A.)

* corresponding author: Henrik Rasmus Andersen e-mail: henrik@ndersen.net

Dose response curve of *Vibrio fischeri* at different time from pure PAA

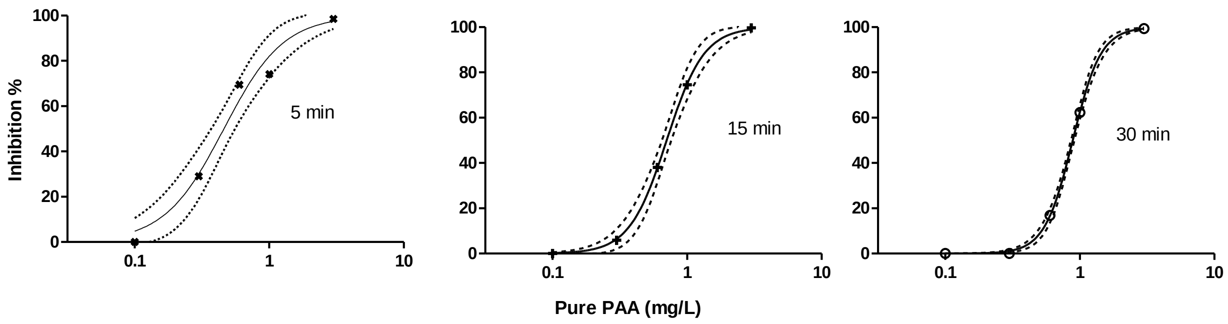

Figure S1: Dose response curve of V. fischeri from pure PAA, at 5, 15 and 30 min exposure time. Dashed lines are 95% confidence interval.

Dose response curve of *Daphnia magna* at different time from pure PAA

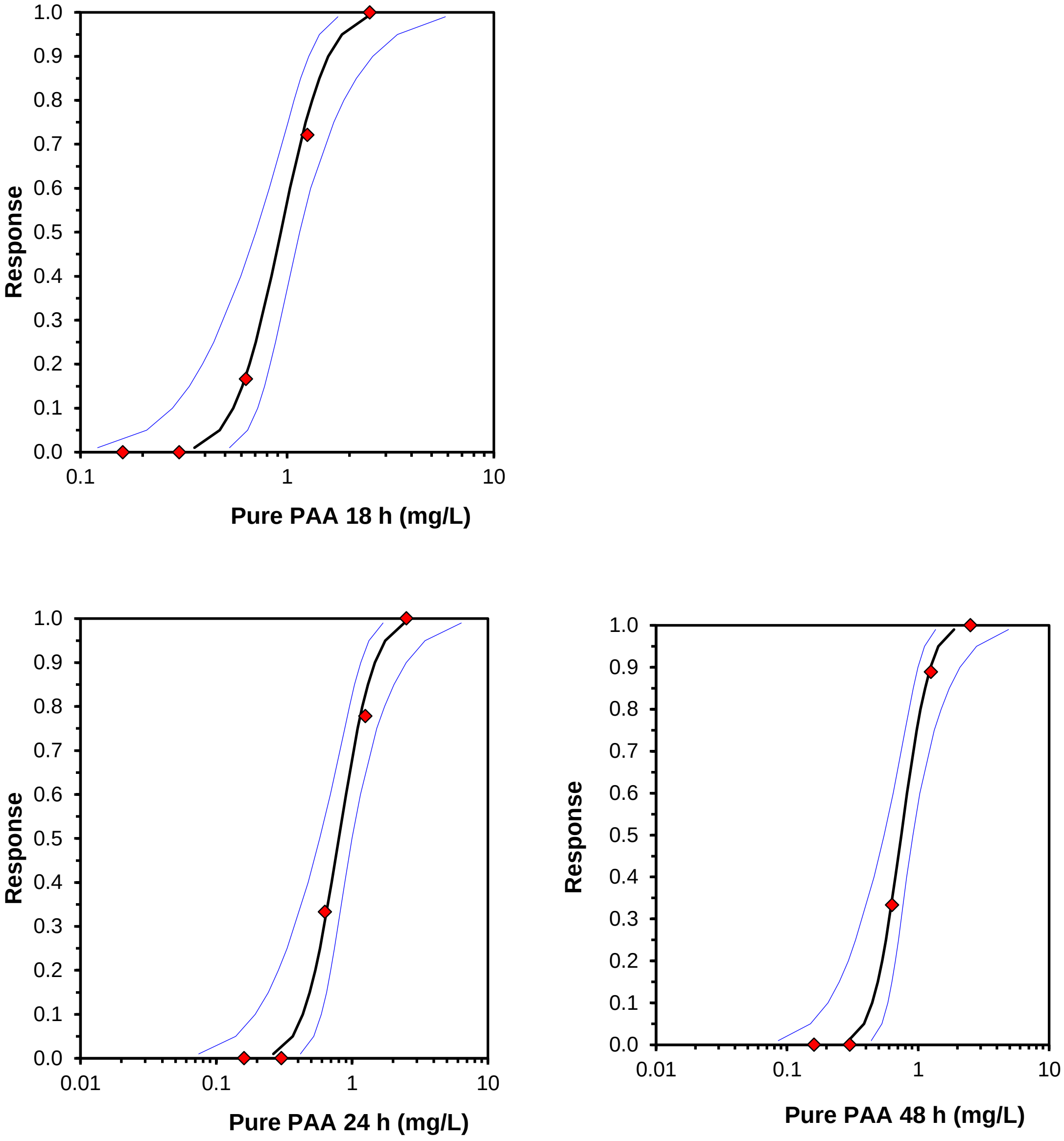

Figure S2: Dose response curve of D. magna from pure PAA at different exposure time. Blue lines are 95% confidence interval.

Dose response curve of *P. subcapitata* at 72 h from pure PAA

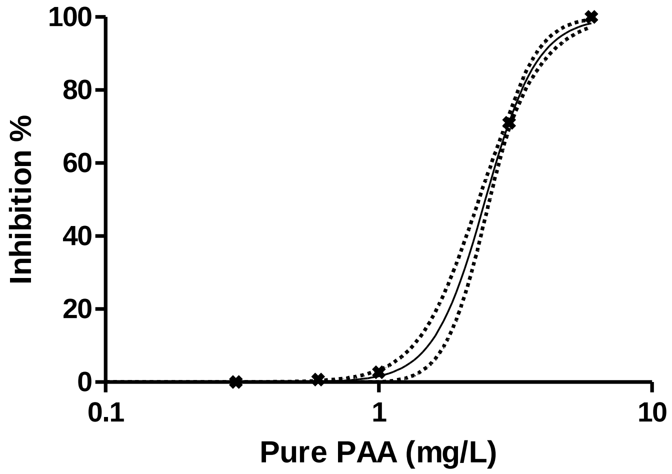

Figure S3: Dose response curve of P. subcapitata from pure PAA at 72 h exposure time. Dashed lines are 95% confidence interval.
